# Giant protists (xenophyophores) function as fish nurseries

**DOI:** 10.1002/ecy.2933

**Published:** 2019-12-17

**Authors:** Lisa A. Levin, Greg W. Rouse

**Affiliations:** ^1^ Scripps Institution of Oceanography University of California, San Diego La Jolla California 92093 USA

During a recent research cruise on the continental margin off Costa Rica, we observed many giant agglutinating Xenophyophoroidea (Foraminifera) on both hard and soft substrates. These fist‐sized protists, which occur only below 400 m, are one of the few groups of organisms limited to the deep ocean (Tendal 1972). Xenophyophores are known to be abundant on sloped topography where there is high particle flux, as many have a morphology designed to trap settling particles that they can feed on or use to form tests (Levin 1991, Levin and Gooday 1992). High abundances and diverse forms have been documented previously in relatively well‐oxygenated parts of the Eastern Pacific Ocean on seamounts (Levin and Thomas 1988), and on abyssal plains (Gooday et al. 2017). Because the elaborate test structures appear to provide substrate, refuge, mating sites and food for deep‐sea invertebrates, xenophyophores occurring on sediments have been recognized as diversity hotspots (Gooday 1984, Levin et al. 1986, Levin and Thomas 1988, Levin 1991). However, to our knowledge, fish have not been known to use xenophyophores as nursery habitat for developing embryos until our research cruise.

**Figure 1:**
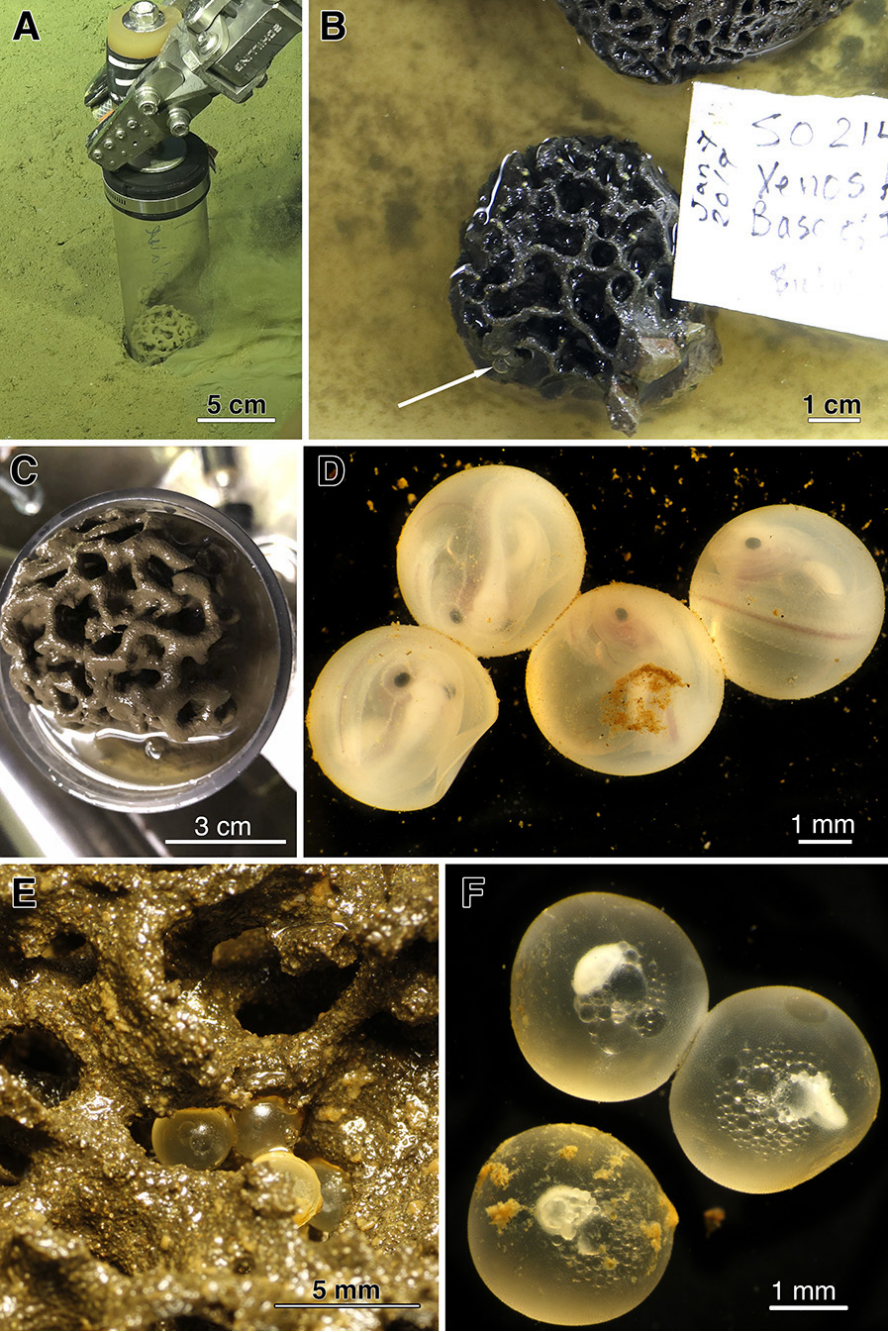
(A, C) Dive S0230 PC 3 *Shinkaiya* sp. collected at 1,902 m, Mound Jaguar 9.656^o^ N 85.881^o ^W; (B, E) *Reticulammina* sp. Dive S0214 Q10, collected at 1866 m, Jaco Scar, 9.117^o ^N 84.842^o ^W; (D) fish embryos in *Shinkaiya* sp. identified as *Acantholiparis* sp. via DNA analysis (GenBank MN509400); (E) fish eggs attached to *Reticulammina* sp. test, identified as *Paraliparis* sp. via DNA analysis (GenBank MN509401); eggs were dead upon discovery, after having been in shipboard incubation experiments for 10 d. (F) Closer view of fish eggs from panel (E).

Collections of Costa Rican xenophyophores from the continental margin were made with the ROV *SuBastian* aboard RV *Falkor* in January 2019, using the ROV manipulator claws for those on rocks and using a pushcore for xenophyophores on sediments (Fig. 1a). Approximately 10 xenophyophores were dissected on board ship to examine associated fauna, from the continental slope and from four nearby seamounts. Here we report the occurrence of snailfish (Liparidae) embryos (Fig. 1d) and eggs (Fig. 1e), attached deep in xenophyophores collected from two sites on the Costa Rican slope at 1,902 m (Mound Jaguar) and 1,866 m (Jaco Scar). Liparidae, a group highly adapted to the deep sea (Gerringer 2019) and well known for their brood‐hiding relationships with invertebrates (Chernova 2014), have never been reported to lay their eggs in any protozoan tests.

Two xenophyophores (Fig. 1b, c) were found to contain fish embryos and eggs, one from hard substrate and one from soft sediments. One 5‐cm diameter xenophyophore from Jaco Scar, tentatively identified based on morphology and test particles as being in the genus *Reticulammina* (Fig. 1b), contained approximately 20 fish eggs (~2.9 mm diameter; Fig. 1f). A 6‐cm diameter xenophyophore, tentatively identified as *Shinkaiya* (Fig. 1c), contained 16 fish embryos (approx. 3.6 mm diameter) (Fig. 1d). Although there is no direct evidence, the intact nature of the xenophyophore tests (Fig. 1b, c) suggest these protists were probably alive at the time of sampling. Representatives from each cluster have been deposited at the Scripps Institution of Oceanography Benthic Invertebrate Collection (SIO‐BIC BI1369 and BI1371).

DNA was extracted from one egg/embryo cluster found in each of the xenophyophores. An ~650 base‐pair fragment of Cytochrome c oxidase subunit I (COI) was then amplified and sequenced for each using standard primers and methods (see Garcia et al. 2019). The sequences (deposited on GenBank, MN509400 and MN509401) were analysed with available COI data on GenBank using BLAST, a maximum likelihood phylogenetic approach (Stamatakis 2014) and also simple pairwise distances (see Garcia et al. 2019; BLAST *available online*).1blast.ncbi.nlm.nih.gov/ Taxon sampling for the phylogenetic analysis was based on the BLAST results against available sequences on GenBank and the studies by Knudsen et al. (2007) and Orr et al. (2019). The egg (SIO‐BIC BI1369; MN509400) sequenced from the Jaco Scar xenophyophore (*Reticulammina*) was less than 3% distant from a series of *Paraliparis* spp. COI sequences on GenBank and in the phylogenetic tree was nested among the sequences from this genus (Appendix S1: Fig. S1). The closest BLAST hit was to some unidentified *Paraliparis* (e.g., GenBank MF956928) that were sequenced from the shelf edge of Pacific Central America (Robertson et al. 2017). The nearest bathyal named *Paraliparis* species are from the Galapagos, where two species have been described at 637 and 710 m (Stein and Chernova 2002), but they have yet to be sequenced. Two species of *Paraliparis* were collected by the *Albatross* off Panama in the 1800s (Chernova et al. 2004).

The embryo sequenced from the *Shinkaiya* sp. xenophyophore (SIO‐BIC BI1371; MN509401) at Mound Jaguar was ~94% identical to a series of liparids in the genera *Acantholiparis* and *Careproctus* and in the phylogenetic analysis formed a weakly supported clade (Appendix S1: Fig. S1) with an *Acantholiparis opercularis* COI sequence (GenBank FJ164243) from off British Columbia (Steinke et al. 2009). *Acantholiparis* has not been previously recorded from the Costa Rica region; the two known species in this genus have their southernmost distributions in Oregon (Grinols 1969) and Northern California (Grinols 1966) at bathyal depths, so this may represent a southern extension of the genus.

We did not observe liparids on the videos recorded during the two ROV dives (S0214 and S0230) on which the xenophyophores were collected. Actual liparid observations made during the same cruise (FK190106) on the Costa Rica margin included a fish similar in appearance to *Careproctus hyaleius* (known from vents in the Eastern Pacific) and an unknown snailfish attached to a lithodid crab (possibly *Careproctus* or *Eknomoliparis*; M. Gerringer, *personal communication*).

Snailfishes have an unusual suite of egg‐depositing behaviors, with an ovipositor that allows them to inject eggs into out of the way places in live animals (called ostracophilia), presumably so the host can supply oxygen or provide protection from predators. These include the gills of lithodid crabs (carcinophilia) (Yau et al. 2000, Gardner et al. 2016), the paragastral cavity of glass sponges (spongiophilia; Chernova 2014), the stalks of octocorals (octocoraphilia; Busby et al. 2006), and the mantle cavity of bivalves (valvatophilia; Andriashev 2003). We can now add xenophyophilia to the list of snailfish brood hiding behaviors. 

Stable isotope signatures of the eggs/embryos were analyzed on a GV Micromass Isoprime continuous flow isotope ratio mass spectrometer (GV CV‐IRMS) at Washington State University. Signatures of snailfish embryos in both xenophyophores indicated they were from a predatory (high trophic level) species from a food web based on photosynthetically derived carbon (rather than chemosynthesis), despite proximity of methane seeps within 30‐50 m at both Jaco Scar and Mound Jaguar. For two *Acantholiparis* sp. embryos in *Shinkaiya,* the δ^13^C signatures were −21.6‰ and −20.3 ‰ and δ^15^N signatures were +17.3‰ and +19.1‰. For *Paraliparis* sp. embryos only one signature was obtained. It was similar: δ^13^C = −20.7‰ and δ^15^N = +16.4‰. The signatures of two xenophyophores collected on dive 0230 when the *Shinkaiya* was sampled (but possibly different species) had remarkably similar carbon signatures (δ^13^C = −20.2‰ and −21.7‰) but significantly lighter δ^15^N (+7.2, +7.4‰).

Xenophyophores have been designated as indicator taxa for Vulnerable Marine Ecosystems (VMEs), which are identified and protected by the Food and Agriculture Organization to reduce ecosystem impacts of bottom fisheries in the deep sea (FAO 2016). VME indicators are organisms that, when observed or caught as bycatch, indicate that fishing may potentially be harmful in an area where VMEs occur. Xenophyophores also appear in the Convention for the Conservation of Antarctic Marine Living Resources (CCAMLR) working definition of VMEs, as set out in CM 22‐06. Morato et al. (2018) developed an assessment scale for 13 VME indicator taxa based on the attributes of uniqueness, function, fragility, life history and structural attributes; each was scored from 1 to 5 and averaged. The scores ranged from 1.48–4.47 with xenophyophores having an indicator score of 3.03, between gorgonians (3.61) and stylasterid corals (2.94). The use of xenophyophores as nursery habitat by at least one group of fishes, the Liparids, though they are not harvested commercially, reinforces the utility of the VME designation for this group of protozoa. The findings of this paper may also have conservation relevance for regions targeted for deep seabed mining where xenophyophores are common, such as in the Clarion Clipperton Zone, (Gooday et al. 2017) or on seamounts (Levin and Thomas 1988).

Snailfishes (Liparidae) are one of the few groups of vertebrates that have become deep‐sea specialists, and are the only fish group to commonly occur in trenches below 7,000 m (Gerringer 2019). Thus, it is interesting to note that they oviposit their embryos into the test structures formed by another group of deep‐sea specialists, Xenophyophoroidea. This association may be limited to snailfishes, as a study of 27 xenophyophores from offshore Eastern Pacific Seamounts (Levin and Thomas 1988) and at least eight other xenophyophores examined from nearby seamounts on the January 2019 RV Falkor cruise did not yield any fish eggs or embryos. However, the presence of metazoan eggs (identity indeterminate) in foraminiferan tests (in the genera *Reophax* and *Saccammina*) was reported as early as 1884 (Rhumbler 1894, Rhumbler 1911 in Gooday 1984). Observations in xenophyophore tests of nematode and sipunculan eggs as well as brooding peracarids and many juvenile ophiuroids and isopods suggest that the tests are commonly used by invertebrates as reproductive or nursery habitat (Gooday, 1984, Levin et al. 1986, Levin 1991). This is the first record of such use by fishes.

The relative proximity of the xenophyophores hosting fish eggs to methane seeps is noteworthy, but there is no evidence that either the xenophyophores or snailfish depositing eggs in the xenophyophores obtain nutrition from the seeps. The frequent occurrence of xenophyophores on the Costa Rica margin at depths of 1,800–2,000 m, and their function as habitat for developing snailfishes, could inform future efforts to conserve these deep‐sea habitats in a region where commercial fishing is widespread.
