# Supplementary material for: Giant protists (xenophyophores) function as fish nurseries
Source: Ecology. 2019 Dec 17;101(4):e02933. doi: 10.1002/ecy.2933 (PMC7341444; doi:10.1002/ecy.2933)
Supplement: Supplementary file 1 [file ECY-101-e02933-s001.pdf]

Supporting Information. Lisa A. Levin and Greg W. Rouse. 2019. Giant protists (xenophyophores) function as fish nurseries. *Ecology*.

Appendix S1

Figure S1. Maximum likelihood tree generated via RaxML (Stamatakis 2014) from *COI* for a range of Liparidae obtained from GenBank and the two eggs sequenced for his study. The model used was GTR+G+I and the numbers on nodes are ML bootstrap scores. Values below 50% for a node are not shown. The taxon sampling and outgroup (Cyclopteridae) are based on based on Kundsen et al. (2007) and Orr et al. (2019).

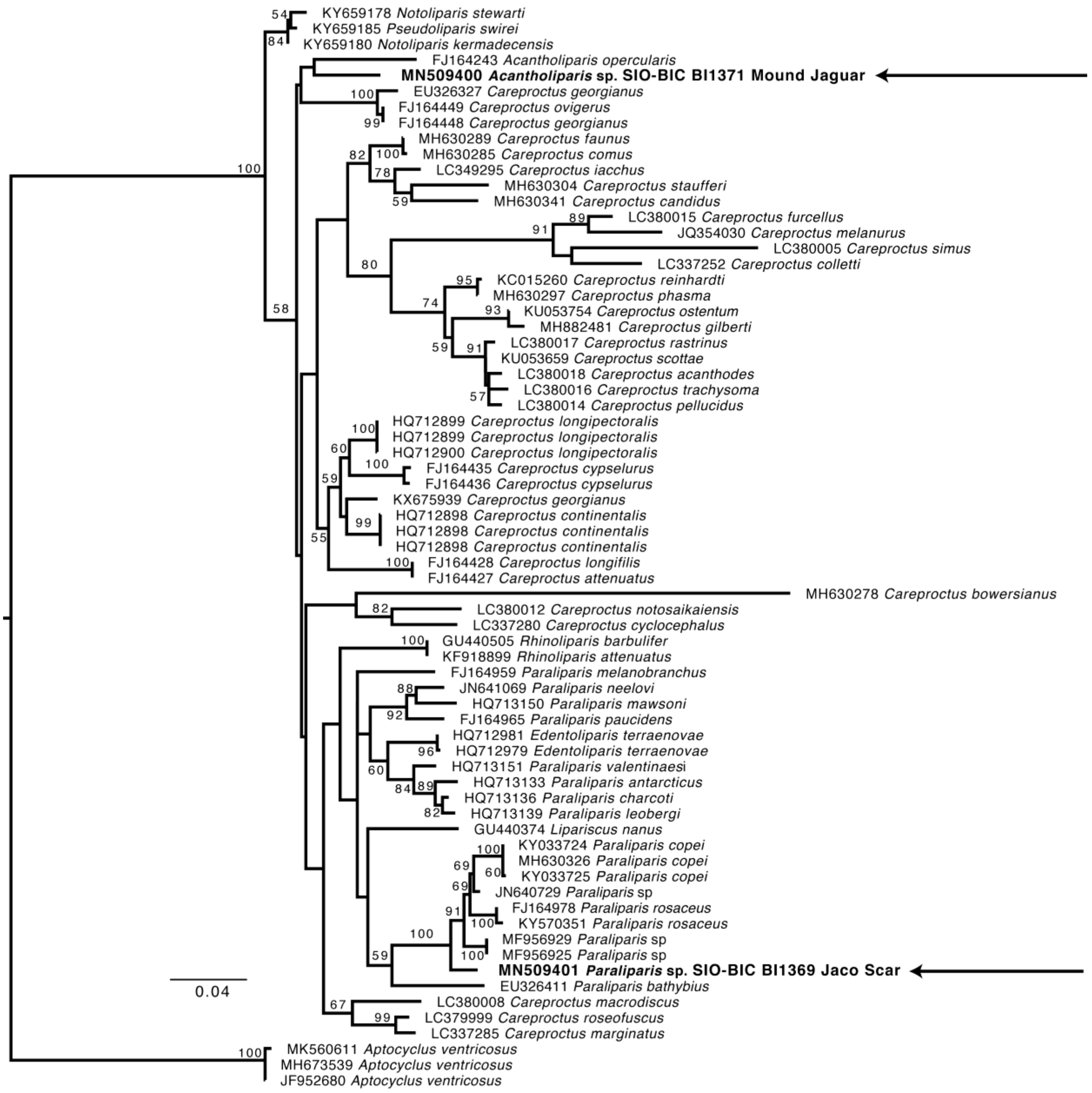

Knudsen, S.W.; Moller, P.R.; Gravlund, P. 2007. Phylogeny of the snailfishes (Teleostei: Liparidae) based on molecular and morphological data. Molecular Phylogenetics and Evolution 44: 649-66.

Orr, James W., I. Spies, D. E. Stevenson, G. C. Longo, Y.i Kai, S. Ghods, and M. Hollowed. 2019. Molecular phylogenetics of snailfishes (Cottoidei: Liparidae) based on MtDNA and RADseq genomic analyses, with comments on selected morphological characters. Zootaxa 4642: 1-79. doi: 10.11646/zootaxa.4642.1.1

Stamatakis, A. 2014. RAxML Version 8: A tool for phylogenetic analysis and post-analysis of large phylogenies. Bioinformatics 30: 1312-13.
